# NEDD4 ameliorates myocardial reperfusion injury by preventing macrophages pyroptosis

**DOI:** 10.1186/s12964-022-01022-y

**Published:** 2023-02-02

**Authors:** Wenjing Sun, Hongquan Lu, Shihua Cui, Shenghui Zhao, Haijia Yu, Huihui song, Qiuyue Ruan, Yabin Zhang, Yingjie Chu, Shujuan Dong

**Affiliations:** 1 Department of Cardiology, Henan Provincial People’s Hospital, Zhengzhou University People’s Hospital, Zhengzhou, 450000 China; 2 Department of Clinical Microbiology, Henan Provincial People’s Hospital, Zhengzhou University People’s Hospital, Zhengzhou, 450000 China; 3 Department of Nuclear Medicine, Third People’s Hospital of Honghe State, Honghe, 661000 China; 4 Department of Cardiology, Dalian Medical University, Dalian, 116000 China; 5 Department of Nephrology, First People’s Hospital of Honghe State, Honghe, 661000 China

**Keywords:** Ischemia/reperfusion, NLRP3 inflammasome, Pyroptosis, NEDD4

## Abstract

**Objectives:**

The inflammatory cascade and cell death post-myocardial ischemia reperfusion (MI/R) are very complex. Despite the understanding that macrophage inflammation has a pivotal role in the pathophysiology of MI/R, the contribution of macrophage inflammatory signals in tailoring the function of vascular endothelium remains unknown.

**Materials and methods:**

In the present study, we analyzed the effects of NEDD4 on the NLRP3 inflammasome activation-mediated pyroptosis in vitro after an acute pro-inflammatory stimulus and in vivo in a MI/R mouse model. TTC and Evan’s blue dye, Thioflavin S, immunohistochemistry staining, and ELISA were performed in wild-type and NEDD4 deficiency mice. THP-1 cells were transfected with si-NEDD4 or si-SF3A2. HEK293T cells were transfected with NEDD4 or SF3A2 overexpression plasmid. ELISA analyzed the inflammatory cytokines in the cell supernatant. The levels of NEDD4, SF3A2, and NLRP3/GSDMD pathway were determined by Western blot. Protein interactions were evaluated by immunoprecipitation. The protein colocalization in cells was monitored using a fluorescence microscope.

**Results:**

NEDD4 inhibited NLRP3 inflammasome activation and pyroptosis in THP-1 cells treated with lipopolysaccharide (LPS) and nigericin (Nig). Mechanistically, NEDD4 maintained the stability of NLRP3 through direct interaction with the SF3A2, whereas the latter association with NLRP3 indirectly interacted with NEDD4 promoting proteasomal degradation of NLRP3. Deletion of NLRP3 expression further inhibited the caspase cascade to induce pyroptosis. Interestingly, inhibiting NLRP3 inflammasome activation in THP-1 cells could prevent cardiac microvascular endothelial cells (CMECs) injury. In addition, NEDD4 deficiency decreased animal survival and increased myocardial infarct size, no-reflow area, and promoted macrophages infiltration post-MI/R.

**Conclusions:**

NEDD4 could be a potential therapeutic target in microvascular injury following myocardial reperfusion.

**Video Abstract**

**Supplementary Information:**

The online version contains supplementary material available at 10.1186/s12964-022-01022-y.

## Introduction

Although the mortality and morbidity of cardiovascular disease (CVD) have declined over the last 50 years, acute myocardial infarction (AMI) is still the main cause of death-related diseases worldwide [1]. Timely reperfusion is an effective strategy for reducing acute myocardial ischemic injury. However, blood flow restoration to the ischemic myocardium may simultaneously lead to reperfusion injuries such as cell death and myocardial dysfunction, resulting in heart failure [2, 3], which still constitutes an urgent clinical problem to be solved. Recent studies have shown that coronary microcirculation is a key target for reducing MI/R [4]. While inflammation and cell death are the hallmarks of myocardial reperfusion injury, the precise mechanisms underlying these effects have not yet been fully elucidated.

Macrophages are innate immune cells that reside and accumulate in the healthy and injured heart [5]. Deregulation of macrophages has been identified as a leading cause of unrestrained inflammation to MI/R and a critical factor in the pathogenesis of microvascular injury following myocardial reperfusion [6]. Macrophages produce cytokines and pro-inflammatory factors that participate in the process of cell death during MI/R [7]. Moreover, dying cells release more inflammatory factors that amplify tissue injury and immune cell infiltration. The endothelial cells lining all blood vessels serve as conduits for blood and tissue nutrient delivery. Nevertheless, the way these factors are released by macrophages' crosstalk with endothelial cells resulting in microvascular dysfunction following myocardial reperfusion, is not fully understood.

NOD-like receptor family, pyrin domain containing 3 (NLRP3) inflammasome has emerged as a key mediator of pathological inflammation in various diseases [8]. Upon activation, NLRP3 inflammasome produces amounts of active cytokines, including IL‑1β and IL-18. It also induces pyroptosis, which is an inflammatory form of programmed cell death that occurs under various pathophysiological conditions [9, 10]. Mechanistically, pyroptosis is the dominant response upon caspase-1/4/5/11 activation, which occurs in macrophage and non-macrophage cells, both of which can cleave Gasdermin-D (GSDMD) to mediate pyroptotic cell death accompanied with excessive inflammatory factor release [11–13]. Previous studies have indicated that suppressing GSDMD cleavage in macrophages can alleviate inflammation and tissue damage in mice [14, 15]. In addition, blocking pro-inflammatory cytokines released from macrophages decreases cardiac inflammation [16]. Our previous work has shown that inhibition of CMECs pyroptosis could reduce MI/R-induced myocardium or microvascular injury [17, 18]. However, whether NLRP3 inflammasome activation induced pyroptosis in macrophages affects the function of CMECs under MI/R remains largely unknown. Furthermore, although the mechanisms for NLRP3 inflammasome activation have been widely studied, its regulatory networks need to be further explored.

Neuronal precursor cell-expressed developmentally down-regulated 4 (NEDD4) is an E3 ubiquitin ligase of the HECT family. It consists of a catalytic C-terminal HECT domain, N-terminal C2 domain, and WW domains responsible for cellular localization and substrate recognition [19]. Previous studies revealed that the NEDD4 exerts an important role in regulating adaptive immunity by mediating the ubiquitination and subsequent degradation of their respective substrates in activated T cells [20]. Yet, the biological function of NEDD4 in the NLRP3 inflammasome activation-induced pyroptosis remains unknown.

In the present study, we investigated a novel role of NEDD4 in regulating NLRP3 inflammasome activation-induced cell death and provided a potential therapeutic target for MI/R caused by NLRP3 inflammasome activation-related pyroptosis in macrophages.

## Materials and methods

### Inclusion criteria

① Male C57BL/6J mice (6 ~ 8 weeks old, weighing 18-22 g); ② Animal model successfully established (ST segment elevation was observed after ligation of the left anterior descending, the anterior wall of the left ventricle becomes pale); ③ The transgenic mice were identified successfully.

### Exclusion criteria

① Female C57BL/6J mice; ② Animal model not successfully established (ST segment elevation was not observed after ligation of the left anterior descending, reperfusion was unsuccessful); ③ Intense bleeding during surgery; ④ The needle cut through the ventricle wall; ⑤ Bradycardia during ligation or reperfusion; ⑥ Pulmonary atelectasis.

### Mice

NEDD4 knockout (KO) mice on C57BL/6 background were generated by Bioray Biotech Co, Ltd (Shanghai, China), using CRISPR/Cas9 technique. NEDD4 KO mice and their representative wild-type control mice were maintained in specific-pathogen-free facility. All animal experiments were carried out in accordance with the National Institute of Health Guide for the Care and Use of Laboratory Animals, with the approval of the Scientific Investigation Board of Medical School of Zhengzhou University (Zhengzhou, Henan Province, China), and the protocol for sample collection was authorized by the Ethics Committee of the of Zhengzhou University People’s Hospital (approval date 2020, code 158). Primers used for genotyping: F: 5′–3′ AGATGCTGCCACTCATTTGC; R: 5′–3′ TCATCCGTGAGG-TCA.

### Ischemia–reperfusion model

Adult male Wild-type (WT) and NEDD4^−/−^C57BL/6J mice (6 ~ 8 weeks old, weighing 18-22 g) used in the study were purchased from Cyagen Biosciences and housed under specific pathogen-free conditions. The mice were anesthetized with 0.1 ml 1% pentobarbital injection Sodium by intraperitoneal. After anesthesia, needle electrodes were inserted subcutaneously in the limbs of the mice and the standard ECG leads were recorded. The ventilator was connected after tracheal intubation with 0.12 mL tidal volume and 120–130 times/min respiratory rate. Subsequently, the hearts were then exposed between the fourth and fifth ribs and the left anterior descending (LAD) coronary artery was ligated 6–0 silk suture. It was released for 6 h after occlusion for 45 min. In sham-operated animals, a silk suture was passed under LAD without ligation.

### Assessment of myocardial infarct size

At 6 h after reperfusion, the region of infarct size was measured by double staining with TTC and Evan’s blue dye which proceeded as previously described [18]. The area at risk (AAR) portion of the LV was stained red and white, whereas the infarct size (IS) was stained white and normal myocardium stained dark blue. The heart slices were photographed digitally. After this procedure, the images were analyzed using Image J, the IS and AAR were calculated as a percentage of the LV.

### Measurement of no‐reflow area

Effect of NEDD4 on no-reflow was observed using the dual-staining as previously described [18]. In brief, at the end of I/R (45 min/6 h), the ligation suture was re-ligatured at the same occlusion location of the LAD. Subsequently, 4%, 0.1 ml thioflavin-S was injected via the inferior vena cava immediately followed by re-ligation of the LAD. Then, 3%, 0.1 ml Evan’s blue was injected via tail vein to stain the non-ischemic myocardium. The heart was harvested and washed to remove excessive Evans blue dye. The heart was isolated, froze on − 80 °C refrigerator and cut into 5–6 slices. The heart slices were exposed to UV light (363 nm) using a UV transilluminator for digital photograph. Areas at risk (AAR, absence of Evans blue staining) and areas with no-reflow (attenuated thioflavin-S fluorescence) were calculated using Image J. No-reflow areas were expressed as percentage of LV.

### LPS and nigericin-induced inflammatory model

WT or NEDD4^−/−^ mice (males, 6–8 weeks old) were i.p. injected with 10 mg/kg LPS for 6 h, and followed by i.p. administrated with Nig for 2 h. Sterile endotoxin-free PBS was used as vehicle control in sham groups. After 8 h mice were killed, and serum levels of IL-1β, TNF-α and GSDMD were measured by ELISA, Inflammatory cells infiltration were performed by immunohistochemical.

### Immunohistochemistry

Briefly, immediately after heart excised, heart tissues with a distance of 0.5 cm from the margin of the heart were fixed in 10% buffered formalin. Afterwards, the specimens were dehydrated with a graded ethanol series and paraffin-embedded. The sections were subsequently incubated with 0.3% hydrogen peroxide in PBS to block endogenous peroxidase activity, and treated with 10 mM citrate buffer (pH 6.0) to retrieve the antigen, followed by rinsing in phosphate buffered saline (PBS). Subsequently, the sections were blocked with 5% goat serum in PBS for 2 h, and incubated overnight at 4 °C with the following primary antibodies: rabbit anti-F4/80 (1:100, Cell Signaling Biotechnology), rabbit anti-CD68 antibody (1:100, Cell Signaling Biotechnology). Immunoreactivities were performed with the labeled streptavidin–biotin peroxidase and visualized using diaminobenzidine tetrahydroc diaminobenzidine tetrahydrochloride (DAB) staining. Captured images were further analyzed by software Image J.

### Cell culture

To obtain mouse primary peritoneal macrophages, C57BL/6J mice (female, 4–6 weeks old) were injected intraperitoneally with 3% Brewer’s thioglycollate broth. Three days later, PEC were harvested and incubated. Two hours later, nonadherent cells were removed and the adherent monolayer cells were used as peritoneal macrophages. Human THP-1, human embryonic kidney (HEK293T) cells, Human cardiac microvascular endothelial cells line (HCMECs) and H9c2 were purchased from the Bei Na Chuan-glian Biotechnology (BNCC, Wuhan, China). The cells were cultured at 37 °C under 5% CO2 in high glucose Dulbecco’s modified Eagle’s medium (DMEM: Hyclone, Logan, UT, USA) or RPMI (Hyclone, Logan, UT, USA) supplemented with 10% fetal bovine serum (BI: Israel, Middle East) and 100 IU/ml penicilin and 100 IU/ml streptomycin (Hyclone, Logan, UT, USA).

### Cell treatment

50 ng/mL Phorbol 12-Myristate 13-Acetate (PMA, Sigma) was added to THP-1 cells for 24 h before inducing macrophage-like phenotype, and lipopolysaccharide (LPS, Sigma) 1 mg/ml (4 h), ATP 5 mM (2 h), nigericin 50 μM (1 h) were applied to stimulate cell inflammation of THP-1; LPS 100 ng/ml (4 h), ATP 5 mM (2 h), nigericin 10 μM (1 h) for mouse primary peritoneal macrophages.

For CMECs and H9c2, briefly, oxygen-serum deprivation injury was occurred by placing cells in a hypoxic mosphere (1% O_2_, 5% CO_2_, 94% N_2_) in the absence of serum medium for 2 h. After 2 h, the medium was exchanged for oxygenated and serum medium, and the culture was incubated at 37 °C for 2 h.

### Enzyme-linked immunosorbent assay (ELISA)

The concentrations of mouse IL-1β, mouse TNF-a and mouse GSDMD were measured using ELISA kits (Abcam, Cambriage, UK) according to the manufacturers’ instructions. Results were normalized by volume for serum samples. All samples were tested at least in triplicate.

### Western blot analysis and immunoprecipitation

Total protein was extracted in RIPA lysis buffer (Solarbio, China) on ice, followed by centrifugation at 12,000 rpm for 15 min to remove cell fragments. Protein concentration was examined by the BSA protein assay (Thermo Fisher, USA). Cell and tissue lysates were separated by SDS-PAGE using a Tris-Glycin system, and the protein bands then transferred to a PVDF membrane. The membrane was blocked with 5% fat-free milk in Tris buffered saline (TBS) for 2 h at room temperature and subsequently incubated overnight at 4 °C with the following primary antibodies: NLRP3 (1:1000, Adipogen), Caspase-1 (1:1000, Adipogen), GSDMD (1:1000, Abcam), C-GSDMD (1:1000, Abcam), SF3A2 (1:1000, Abclonal), NEDD4 (1:1000, Proteins) and β-actin (1:5000, Abclonal). After washing three times with TBST, membranes were incubated with horseradish peroxidase (HRP)-conjugated IgG (goat anti-rabbit secondary antibody and goat anti-mouse) secondary antibody for 2 h at room temperature. Proteins were visualized by ECL procedure (Amersham Imager 600). The expression of target proteins was standardized by β-actin.

For immunoprecipitation (IP), cells were lysed with Cell Lysis Buffer (Absin, China) on ice for 15 min. Cell lysates were add with protein A/G-magnetic beads (Santa cruz Biotechnology, USA) for specific antibody and the anti-Flag-beads, anti-Myc-beads for NEDD4, NLRP3 and SF3A2 to incubate with rocking overnight at 4 °C. The immunocomplexes were harvested next day and washed three times with IP lysis buffer. Proteins combined with the beads were eluted with SDS/PAGE sample buffer and subsequently boiled for western blot analysis.

### Immunofluorescence

For cytofluorescense staining, cells grown on glass coverslips were fixed in 4% paraformaldehyde for 20 min and permeabilized in 0.1% Triton X-100 for 15 min. Cells on coverslips were incubated with normal goat serum for 2 h. After extensive washing in PBS, cells were incubated with primary mouse anti-NLRP3 (1:200, AdipoGen) and rabbit anti-SF3A2 (1:200, Abclonal), rabbit anti-NEDD4 (1:200, Proteintech), rabbit anti-GSDMD (1:200, Abcam) antibody overnight at 4 °C. After three washes with PBS, cells were incubated with secondary FITC-conjugated anti-ribbit IgG or FITC anti-rabbit IgG anti-body for an additional 2 h at room temperature. Stained cells were photographed under a fluorescence microscope (Olympus, Tokyo, Japan) with the B-2A (EX: 450–490, DM: 505, BA: 520) and G-2A (EX: 510–560, DM: 575, BA: 590) filters. The fluorescence of cells was quantified using ImageJ software (version 1.37; National Institutes of Health, Bethesda, MD, USA).

### Plasmids transfection

Expression vectors for HA-Ub plasmid were gift from Dr wei Zhao (Shan Dong University, Shandong, China). Flag-NEDD4, Myc-NLRP3 and Myc-SF3A2 plasmids were purchased from Stratagene. Plasmids were transiently transfected into HEK293T cells or CMECs with lip3000 according to the manufacturer’s instructions.

### Statistical analysis

All numerical data were presented as individual data points alongside means and standard deviations. Statistical analysis was performed using GraphPad Prism 9. Kolmogorov–Smirnov test was used to test whether the value comes from a normal distribution. Differences between groups were assessed by *student’s t*-tests (one measured variable) or by a two-way ANOVA with Bonferroni post hoc testing. *P* < 0.05 was considered statistically significant. Survival data from the in vivo experiments were analyzed by a log-rank test performed on curves generated by GraphPad Prism 9.0.

## Results

### NEDD4 alleviates myocardial reperfusion injury in mice

**Fig. 1:**
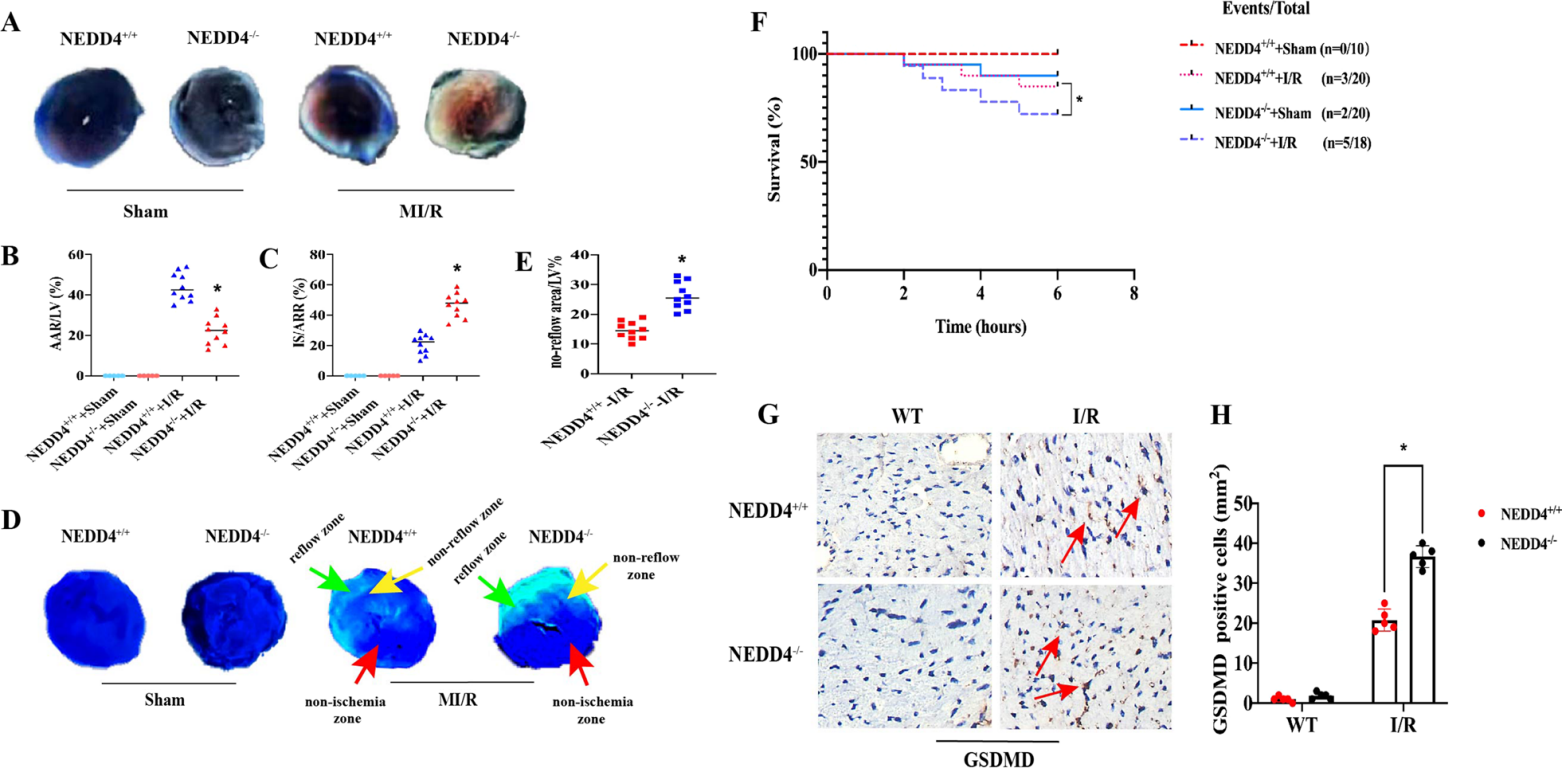
NEDD4 alleviates myocardial reperfusion injury in mice. **A** Representative TTC-Evan’s Blue stained sections of heart from each group. Brick red-stained area represents the area at risk (AAR), whereas the white area indicates the infarcted size (IS). **B**, **C** Ratio of AAR/LV, IS/ARR. Data are expressed as mean ± SD (n=10 for each). *P < 0.05 vs. NEDD4^+/+^+I/R group. **D** Representative cross-sectional images of the left ventricular (LV) showing no-reflow regions indicated by the absence of thioflavin-S fluorescence within the ischemic area that consists of both no-reflow (yellow arrows) and reflow zones (green arrows) following IR. **E** Ratio of no reflow area/LV. Data are expressed as mean ± SD (n=10 for each). *P < 0.05 vs. NEDD4^+/+^+I/R group. **F** Survival analysis for mice subjected to 45 min ischemia and following 6 h perfusion (*P < 0.05 vs. NEDD4^+/+^+I/R group, log-rank test for survival analysis). **G** Effect of NEDD4 on cell pyroptosis. **H** Quantitative analysis of pyroptosis cells was performed. *P < 0.05 vs. NEDD4^+/+^+I/R group

NEDD4^−/−^ decreased animal survival and increased the levels of GSDMD (Fig. 1F) compared to the NEDD4^+/+^ group after MI/R. Figure 1A shows the effect of NEDD4^−/−^ on the myocardial infarct size at 6 h after reperfusion. The ratio of myocardial infarct size to the area at risk (AAR) in the MI/R group was larger than that of the Sham group (Fig. 1C, P < 0.05). However, the ratio of infarct size to AAR in the NEDD4^−/−^-I/R group was larger than that of the NEDD4^+/+^-I/R group (Fig. 1A). The AAR as the percentage of the LV (AAR/LV) in the NEDD4^−/−^-I/R group was significantly increased in comparison with WT-I/R group (Fig. 1B, P < 0.05).

The microvascular damage was evaluated by no-reflow phenomenal. No-reflow area induced by I/R was significantly enlarged in NEDD4^−/−^ group compared to the NEDD4^+/+^ group (Fig. 1D, E, P < 0.05). Furthermore, it was essential to establish whether the up-regulation of GSDMD was observed in the heart following MI/R. In order to address this issue, the expression of GSDMD was examined in NEDD4^+/+^ and NEDD4^−/−^ mice by immunohistochemistry. As expected, NEDD4 deficiency greatly enhanced GSDMD protein expression in the heart post-myocardial reperfusion (Fig. 1G, H, P < 0.05).

**Fig. 2:**
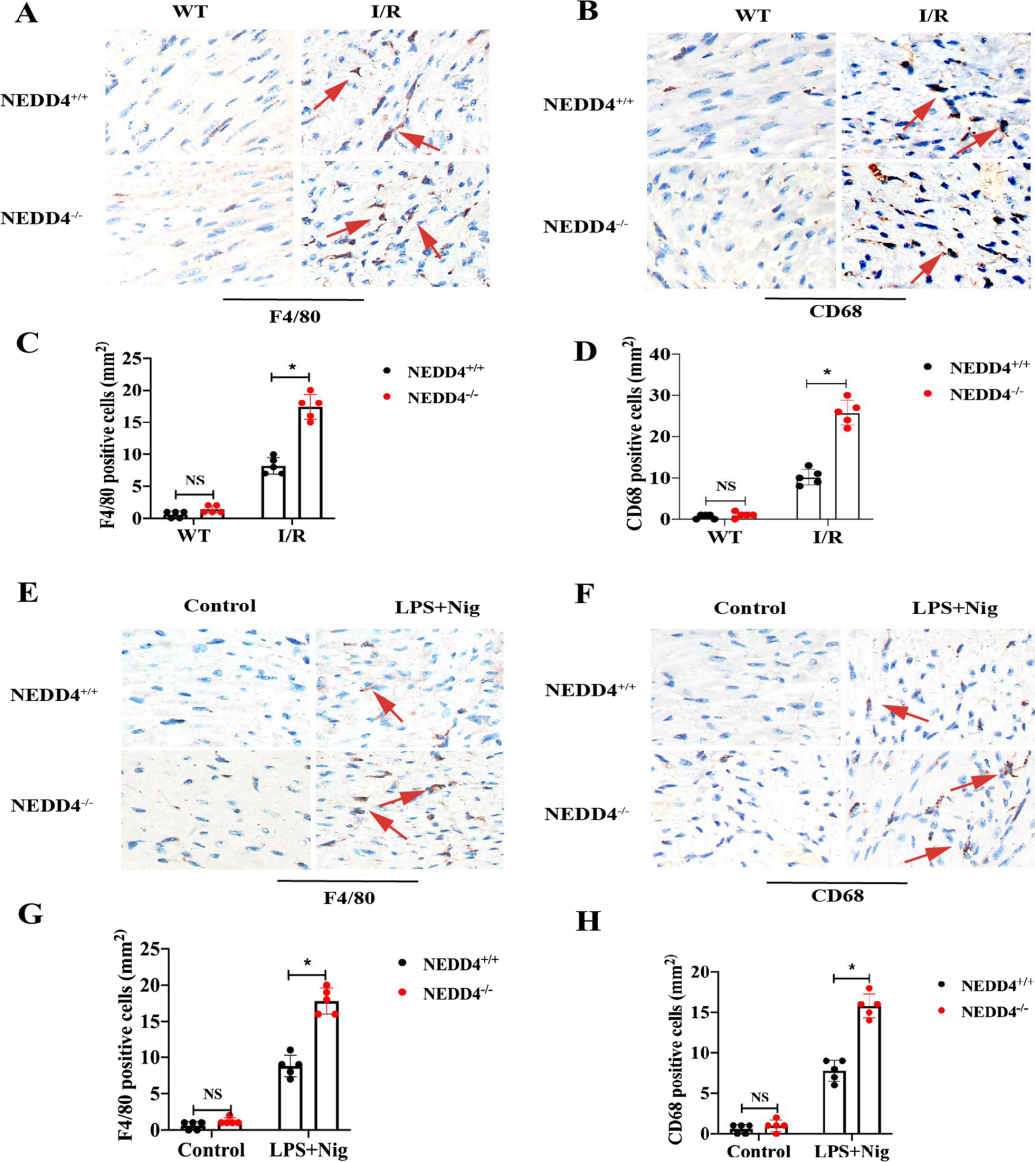
Effect of NEDD4 on inflammatory cells infiltration in mice. **A**, **B** F4/80+ and CD68+cells were up-regulated in NEDD4^−/−^ mice heart at 6 h after MI/R. **C**, **D** The number of F4/80+ and CD68+ cells were quantified. **E**, **F** NEDD4^−/−^ promotes macrophage infiltration in the heart at 8 h after pro-inflammatory stimulus. **G**, **H** The percentage of F4/80+ and CD68+ cells were counted in the heart

Our data also showed that NEDD4^−/−^ increased F4/80^+^ and CD68 macrophage infiltration in the heart compared to the NEDD4^+/+^ group after MI/R (Fig. 2A–D, *P* < 0.05). Immunohistochemistry analysis was also performed for evaluation of the infiltration of macrophages in NEDD4^+/+^ and NEDD4^−/−^ mice, primed with LPS and followed by stimulation with Nig. Consistent with the above results, LPS/Nig stimulated significant infiltration of macrophages in the heart, which was worsened in NEDD4^−/−^mice (Fig. 2E–H, *P* < 0.05).

### Effect of NLRP3 inflammasome activation and pyroptosis in macrophages on CMECs

**Fig. 3:**
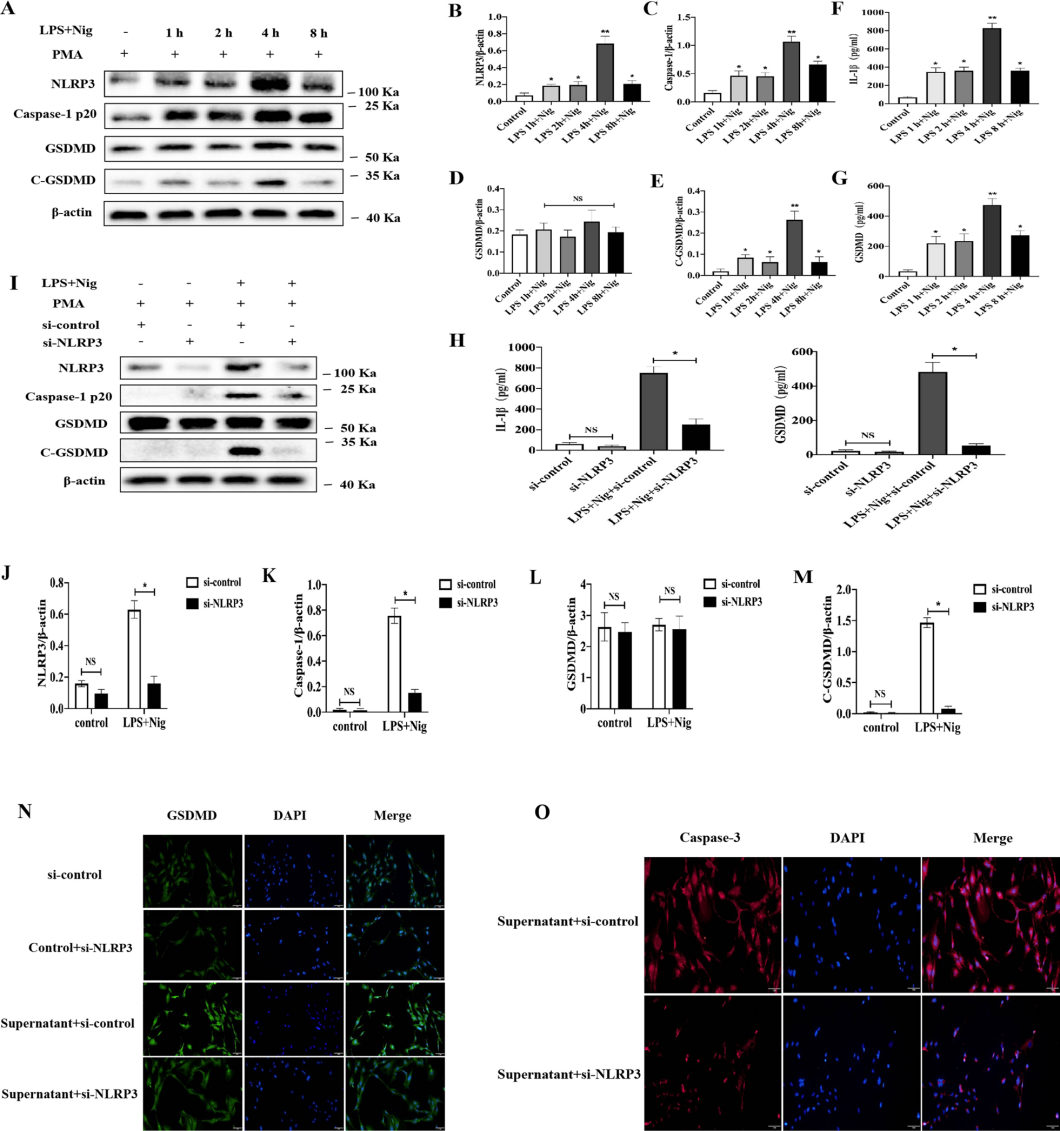
Effect of NLRP3 inflammasome activation in macrophages on CMECs. **A**–**E** Immunoblot analysis and quantification by densitometry of NLRP3, Caspase-1 (p20), GSDMD and C-GSDMD protein in lysates (n=3). **F**–**G** ELISA analysis supernatants of IL-1β and GSDMD in THP-1 cells treated with LPS (1μg/ml), and followed by stimulation with10 μM nigericin (n=3). **H**–**L** Immunoblot analysis and quantification by densitometry of NLRP3, Caspase-1 (p20), GSDMD and C-GSDMD protein in lysates from THP-1 cells transfected with si-NLRP3 (n=3). **M** ELISA analysis supernatants of IL-1β and GSDMD in THP-1 cells treated with si-NLRP3. **N**–**O** Immunofluorescence staining for GSDMD and caspase-3 in CMECs at 12 h after conditioned medium stimulation from LPS/Nig-activated or control THP-1 cells with or without transfected with si-NLRP3 (n=5). Scar bar=20μm

Our previous studies revealed that hypoxia-reoxygenation (H/R) induces NLRP3/caspase-1 or caspase-4 mediated pyroptosis in CMECs [17, 18]. Macrophages have an important role in the pathological process of MI/R. However, it remains unclear whether NLRP3 inflammasome activation-mediated macrophages release inflammatory factors or pyroptosis, thus further amplifying CMECs damage. Firstly, to confirm the effect of GSDMD on human THP-1 cells pyroptosis, we examined the NLRP3 inflammasome activation and expression of GSDMD primed by LPS and then treated by NLRP3 inflammasome activator nigericin (Nig). The expression of NLRP3, caspase-1 (p20), and C-GSDMD was significantly increased in a time-dependent manner, peaking at 4 h after LPS and Nig treatment (Fig. 3A–E). The serum concentrations of IL-1b and GSDMD in supernatants from THP-1 cells were enhanced, primed with LPS at different times, and followed by stimulation with Nig for 1 h (Fig. 3H).

Next small-interfering RNA (si-RNA) was used to diminish NLRP3 expression in human THP-1 cells. Knockdown of NLRP3 expression markedly decreased the expression of NLRP3, caspase-1 (p20), and C-GSDMD (Fig. 3I–M).

Then, we investigated whether human THP-1 cells release pro-inflammatory or death factor-induced CMECs pyroptosis. To further demonstrate the effect of macrophages on CMECs, we collected conditioned medium from either LPS/Nig-activated or control THP-1cells, which were added to CMECs. After 12 h, immunofluorescence was performed for CMECs. The results showed that the expression of GSDMD and caspase-3 was higher in human THP-1 cells supernatant treated by LPS and Nig than in those without treatment. Knockdown of NLPR3 expression significantly decreased the GSDMD and caspase-3 expression in CMECs (Fig. 3O–N).

### NEDD4 inhibits NLRP3 inflammasome activation and pyroptosis in macrophages

**Fig. 4:**
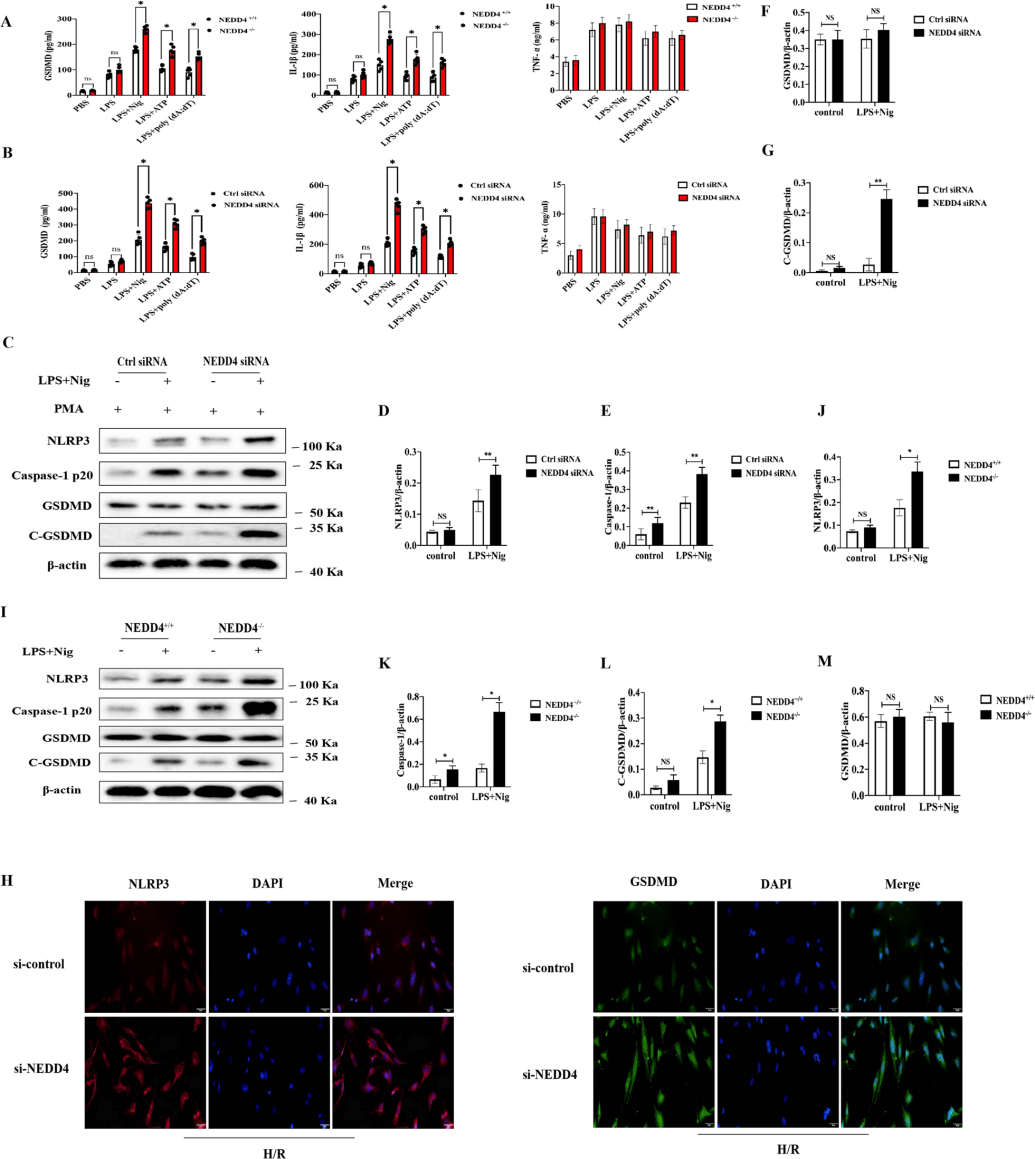
NEDD4 negatively regulates NLRP3 activation and pyroptosis. **A** ELISA of GSDMD, IL-1β and TNF-α in supernatants from NEDD4^+/+^ or NEDD4^−/−^ mouse treated with LPS for 6 h, and followed by stimulation with ATP, Nig. or poly (dA:dT) for 2 h. Data are expressed as mean ± SD (n = 5). *P < 0.05 vs. NEDD4^+/+^ group. **B** ELISA of GSDMD, IL-1β and TNF-α in supernatants from mouse peritoneal macrophages silenced of NEDD4, primed with LPS for 6 h, and followed by stimulation with ATP, Nig. and poly (dA:dT) for 2 h. Data are expressed as mean ± SD (n = 5). *P < 0.05 vs. ctrl siRNA group. **C**–**G** Immunoblot analysis and quantification by densitometry of NLRP3, Caspase-1 (p20), GSDMD and C-GSDMD protein in lysates from THP-1 cells transfected with si-NEDD4, primed with LPS 4 h, and followed by stimulation with Nig for 1 h. Data are expressed as mean ± SD (n = 3). **P < 0.05 vs. ctrl siRNA group. **H** Immunofluorescence staining for NLRP3 in CMECs transfected with or without si-NEDD4.** I**-**M** Immunoblot analysis and quantification by densitometry of NLRP3, Caspase-1 (p20), GSDMD and C-GSDMD protein in lysates from mouse peritoneal macrophages silenced of NEDD4, primed with LPS 4 h, and followed by stimulation with Nig for 1 h. Data are expressed as mean ± SD (n = 3). *P < 0.05 vs. NEDD4^+/+^ group

In order to investigate whether modulation of NEDD4 levels in macrophages influences NLRP3 inflammasome activation and pyroptosis, we diminished the NEDD4 expression in human THP-1 cells using siRNA and found that knockdown of NEDD4 expression markedly increased ATP and Nig induced caspase-1 cleavage and the level of GSDMD (Fig. 4C–G, *P* < 0.05). Notably, similar results were also observed in NEDD4-deficient CMECs after H/R (Fig. 4H). We then investigated whether NEDD4 deficiency could regulate this induction of IL-1β, finding that IL-1β and GSDMD secretion was significantly increased in NEDD4 silenced THP-1 cells primed by LPS and then treated by NLRP3 activators such as ATP, nigericin, or poly (dA:dT) (Fig. 4B). However, TNF-α secretion was not influenced by NEDD4 knockdown (Fig. 3, *P* < 0.05).

To confirm the inhibitory role of NEDD4 in IL-1β and GSDMD secretion, the effects of NEDD4 deficiency on IL-1β and GSDMD expression in mice serum were observed. ATP and Nig stimulated IL-1β, and GSDMD secretion by LPS-primed NEDD4-deficient mice, were significantly increased (Fig. 4A, *P* < 0.05).

### NEDD4 promotes proteasomal degradation of NLRP3

Neural precursor cell-expressed developmentally down-regulated gene 4 (NEDD4) is one of the HECT E3s and is characterized by an N-terminal C2 domain, 3–4 WW repeats, and a C-terminal HECT domain. NEDD4 exerts important regulatory roles in the negative regulation of innate immune responses by promoting the degradation of their respective substrates through the ubiquitin–proteasome pathway. However, the function of NEDD4 in NLRP3 inflammasome-medicated pyroptosis remains unknown.

**Fig. 5:**
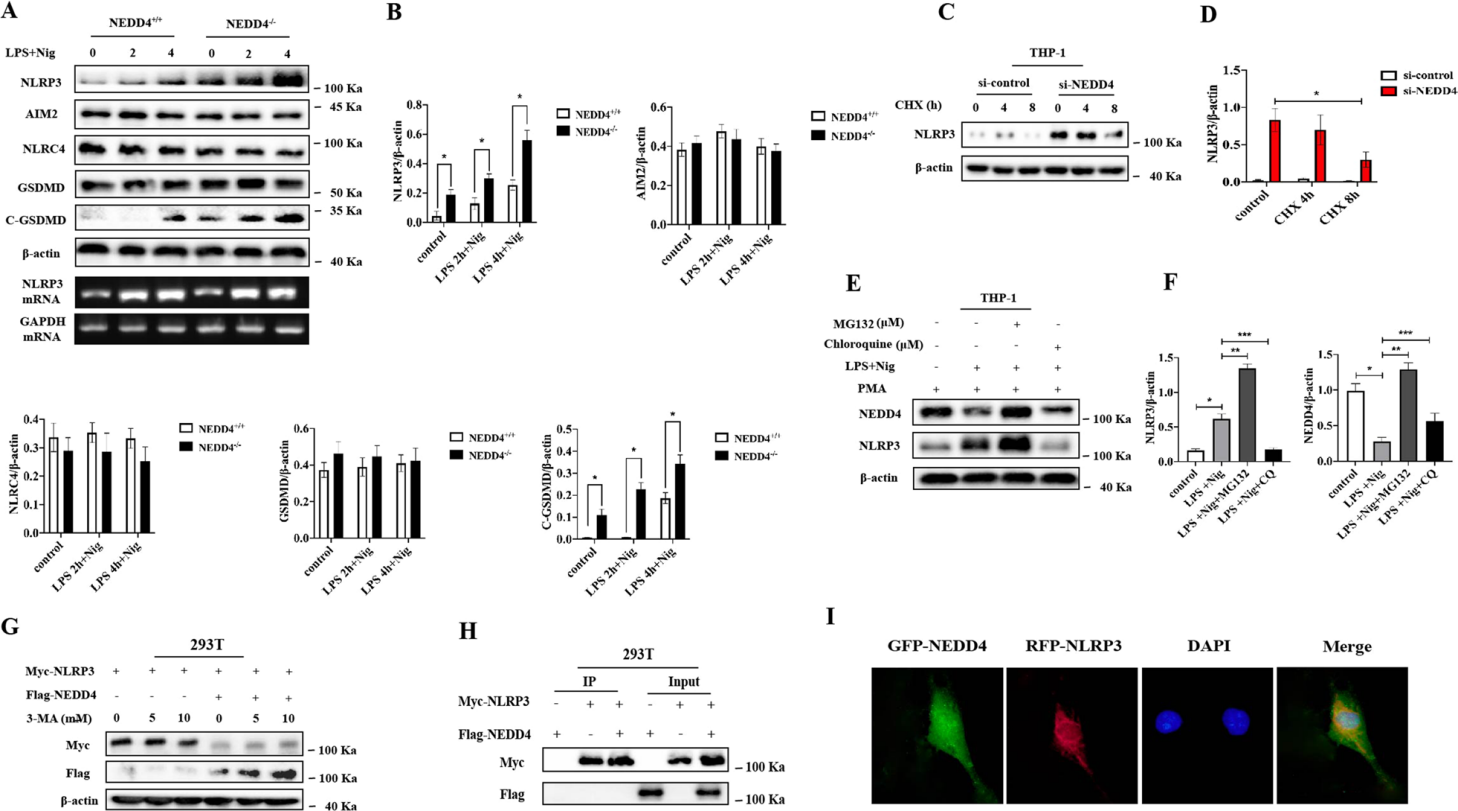
NEDD4 indirectly promotes proteasomal degradation of NLRP3. **A**, **B** Immunoblot analysis and quantification by densitometry of NLRP3, NLRC4, AIM2, GSDMD and C-GSDMD protein or RT-PCR analysis of mouse peritoneal macrophages from NEDD4^+/+^ or NEDD4^−/−^ mouse, and then stimulated with LPS/Nig at different times. Data are expressed as mean ± SD (n = 3). *P < 0.05 vs. NEDD4+/+group. **C**, **D** Immunoblot analysis and quantification by densitometry of NLRP3 from THP-1 cells silenced of NEDD4, and then treated for various times with cycloheximide (CHX). Data are expressed as mean ± SD (n = 3). *P < 0.05 vs. ctrl siRNA group. **E**, **F** Immunoblot analysis of extracts from THP-1 cells stimulated with LPS for 4 h, and followed by stimulation with Nig for 1 h. Then treated with MG-132 and chloroquine (CQ) for 4 h. Data are expressed as mean ± SD (n = 3). *P < 0.05 vs. LPS+Nig, **P < 0.05 vs. LPS+Nig+MG132, ***P < 0.05 vs. LPS+Nig+CQ. **G** Immunoblot analysis of extracts from HEK293T cells transfected with Myc-NLRP3 and Flag-NEDD4 expression plasmid then treated with 3-MA. **H** HEK293T cells were transfected with NEDD4 and NLRP3, and cell lysates were immunoprecipitated with anti-Flag or anti-Myc antibody. The immunocomplex was analyzed by immunoblotting. **I** CMECs transfected with GFP-NEDD4 and RFC-NLRP3 were fixed and incubated with a secondary antibody conjugated to Alexa Fluor 594 and Alexa Fluor 488. Non-colocalization between NEDD4 and NLRP3 was examined by fluorescence microscope

To confirm whether NEDD4 could inhibit NLRP3 inflammasome activation by promoting protein degradation of inflammasome components, the expression of NLRP3 inflammasome components and GSDMD was examined in mouse peritoneal macrophages following LPS/Nig stimulation in a time-dependent manner, revealing that NEDD4 knockdown greatly increased NLRP3 and C-GSDMD expression in mouse peritoneal macrophages (Fig. 5A, B). However, NEDD4 deficiency could not increase AIM2 and NLRC4 expression (Fig. 5A, B). Interestingly, NEDD4 knockdown failed to affect NLRP3 mRNA expression (Fig. 5A), suggesting that NEDD4 inhibits NLRP3 expression at the post-transcriptional level.

Given the functions of NEDD4 in promoting the degradation of its target proteins, we speculated that NEDD4 might inhibit NLRP3 protein expression by promoting its degradation. Our results showed that NEDD4 deficiency inhibited NLRP3 protein degradation (Fig. 5C, D) and that NEDD4 suppressed NLRP3 expression by promoting its protein degradation. Next, we explored the degradation pathway of NLRP3 mediated by NEDD4. NLRP3 degradation was reversed by the proteasome inhibitor MG-132, but not the lysosome inhibitor chloroquine or the autophagy inhibitor 3-MA (Fig. 5E, F), thus suggesting that NEDD4 inhibits the proteasomal degradation of NLRP3. To further investigate how NEDD4 promotes NLRP3 degradation, we examined the interaction between NEDD4 and NLRP3. Interestingly, we found no direct interaction of NEDD4 with NLRP3 (Fig. 5H–I).

### SF3A2 promotes NLRP3 inflammasome activation and pyroptosis

**Fig. 6:**
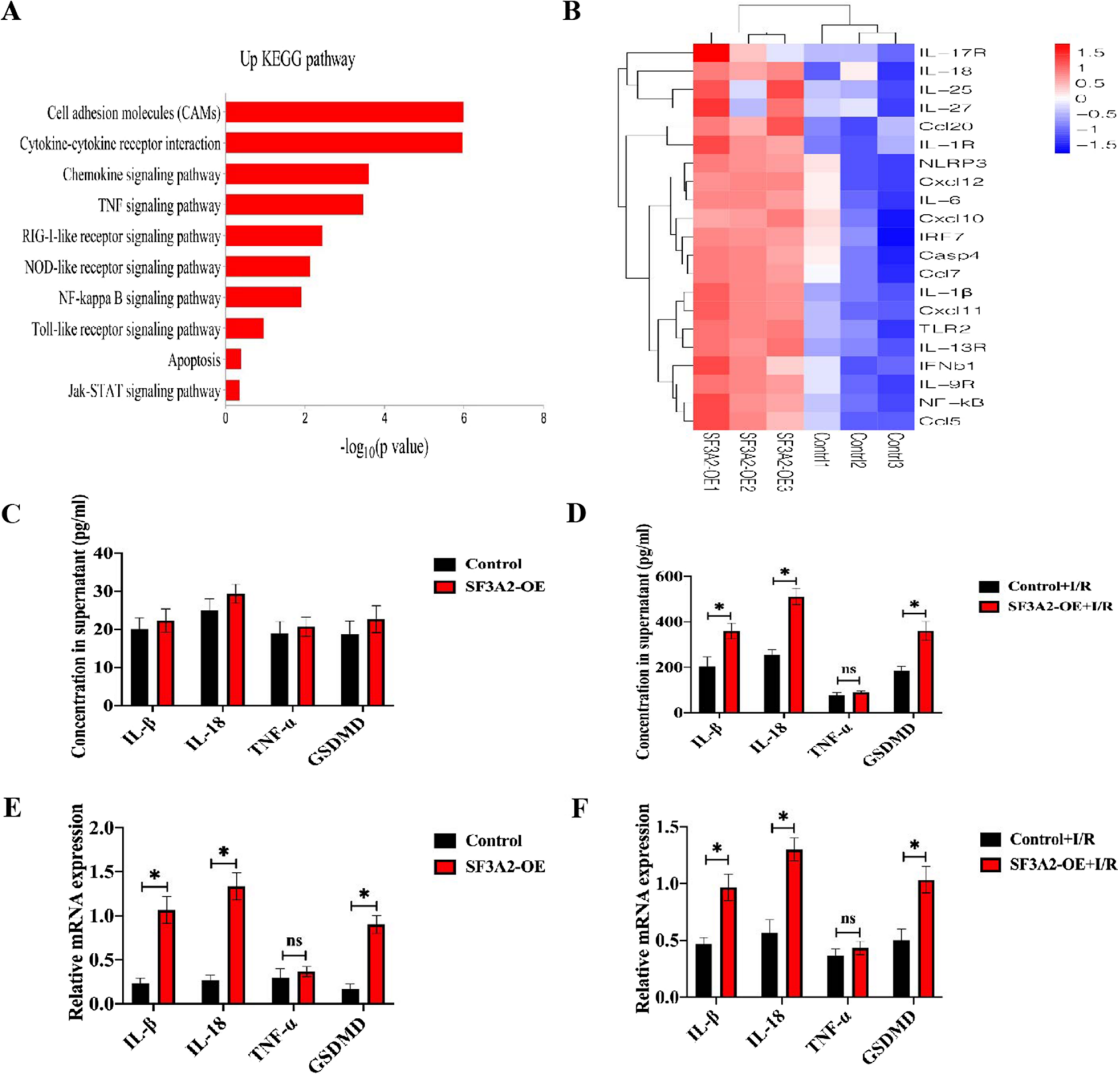
Impaired inflammatory response with SF3A2 overexpression after I/R. **A** Heat mapping of the top 20 ranked (by p value and fold-change) genes that were differentially expressed in H9c2 cells transfected with SF3A2 overexpression plasmid, subjected to I/R. **B** Kyoto Encyclopedia of Genes and Genomes (KEGG) pathway analysis of differentially expressed genes in H9c2 cells transfected with SF3A2 overexpression plasmid subjected to I/R. **C**–**F** mRNA gene expression analysis of inflammatory gene was verified in cell lysates and supernatants from H9c2 cells subjected to I/R, transfected with vector or SF3A2 overexpression plasmid. Data are expressed as mean ± SD (n = 3). *P < 0.05 vs. control+ I/R group

A previous study indicated SF3A2 as new susceptibility loci for MI [21]. However, the role of SF3A2 in the pathological development of MI/R remains unclear. To determine whether SF3A2 contributes to the myocardium or endothelium injury, we performed RNA-Seq analysis of SF3A2 after H9c2 transfected with Flag-SF3A2, finding obvious upregulation of genes involved in pathways associated with cytokine–cytokine receptor interaction, chemokine signaling pathway, NOD-like receptor signaling pathway and apoptosis (Fig. 6A). The key candidates included genes encoding NLRP3 and related cytokine, cytokine receptor, chemokine, chemotaxis and immunity (Fig. 6B). Qualitative and quantitative analysis of IL-1β, IL-18 and GSDMD were markedly increased in both the supernatants and mRNA from H9c2 cells transfected with overexpression SF3A2, while the level of TNF-α did not change after I/R (Fig. 6C, F), thus indicating that SF3A2 may be involved in the regulation of NLRP3 inflammasome medicated inflammatory response and pyroptosis.

**Fig. 7:**
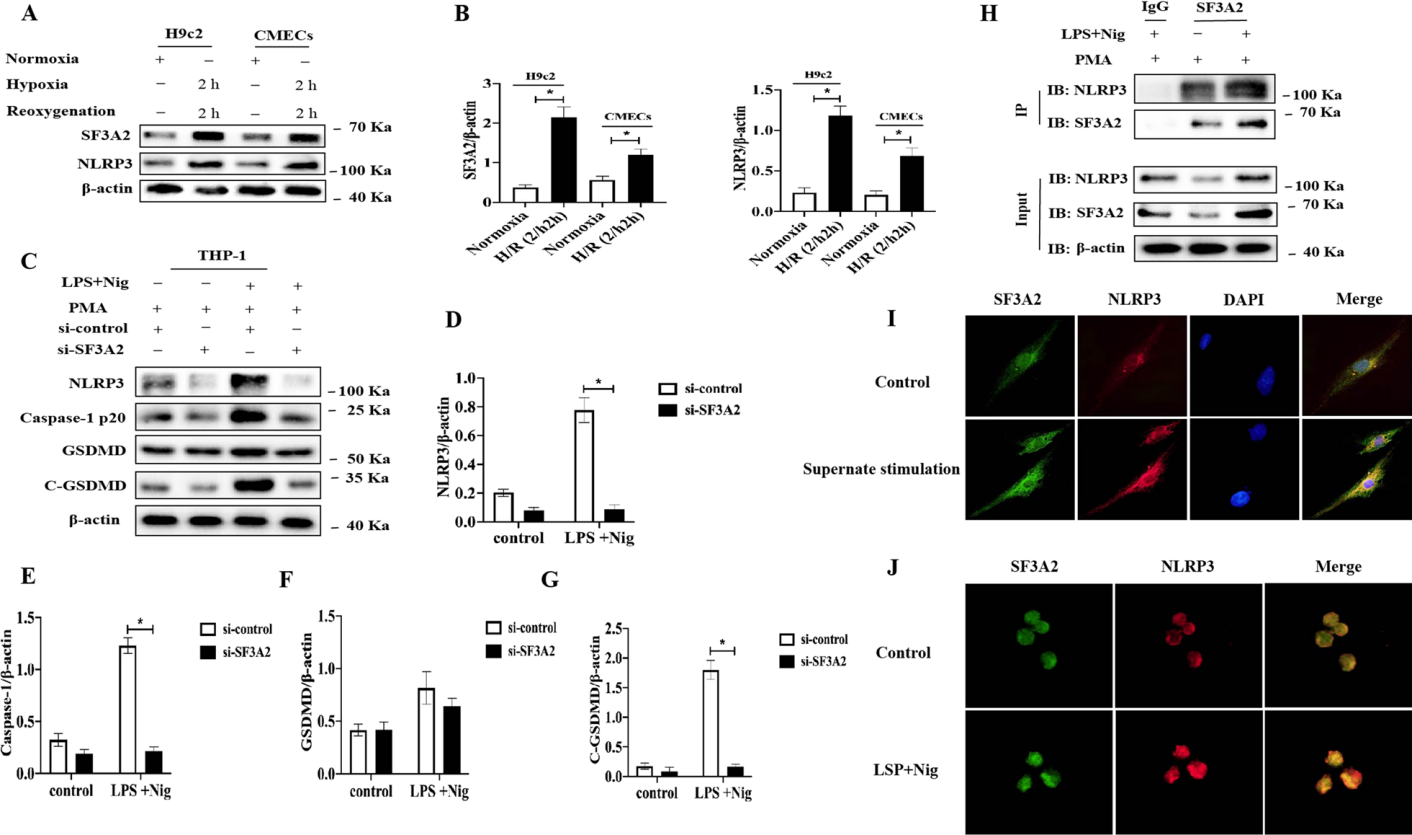
SF3A2 positively regulates NLRP3 activation and pyroptosis. **A**, **B** Immunoblot analysis and quantification by densitometry of SF3A2 and NLRP3 protein in both H9c2 and CMECs cultured under normoxia or subjected to hypoxia/reoxygenation (H/R) (2/2 h). Data are expressed as mean ± SD (n = 3). *P <0.05 vs. normoxia. **C**–**G** Immunoblot analysis and quantification by densitometry of NLRP3, Caspase-1 (p20), GSDMD and C-GSDMD protein in lysates from THP-1 cells transfected with si-SF3A2, primed with LPS 4 h, and followed by stimulation with Nig for 1 h. Data are expressed as mean ± SD (n = 3). *P<0.05 vs. si-control group. **H** Lysates of THP-1 cells treated with LPS for 4 h, and followed by stimulation with Nig for 1 h were immunoprecipitated with anti-SF3A2 antibody. **I** Immunofluorescence staining for SF3A2 and NLRP3 colocalization in CMECs after conditioned medium stimulation from LPS/Nig-activated or control THP-1 cells. **J** Immunofluorescence staining for SF3A2 and NLRP3 colocalization in THP-1 cells with or without stimulation with LPS and Nig

We next examined whether SF3A2 can be activated following H/R induced in CMECs and H9c2 cells. The SF3A2 content of CMECs and H9c2 cells from normoxia and H/R were assessed by Western blotting: the expression of SF3A2 was elevated in the H/R group compared to normoxia (Fig. 7A, B). To further investigate the association of SF3A2 with NLRP3 inflammasome activation-induced pyroptosis in macrophages, we diminished the SF3A2 expression in THP-1 cells using si-SF3A2. Our results showed that the knockdown of SF3A2 expression markedly down-regulated the expression of NLRP3, capase-1 (p20), and C-GSDMD with LPS and Nig stimulation in THP-1 cells (Fig. 7C–G). To further investigate the mechanisms through which SF3A2 promotes the NLRP3 inflammasome activation, we tested whether SF3A2 interacted with NLRP3 and observed strong interaction of SF3A2 with NLRP3 by immunoprecipitation (IP) with resting and LPS/Nig-stimulated THP-1 cells (Fig. 7H). Consistently, immunofluorescence also demonstrated the colocalization between SF3A2 and NLRP3 in both CMECs and THP-1 cells (Fig. 7I, J). Taken together, these data indicated that SF3A2 could directly interact with NLRP3.

### NEDD4 inhibits NLRP3 inflammasome activation and pyroptosis by ubiquitination SF3A2

**Fig. 8:**
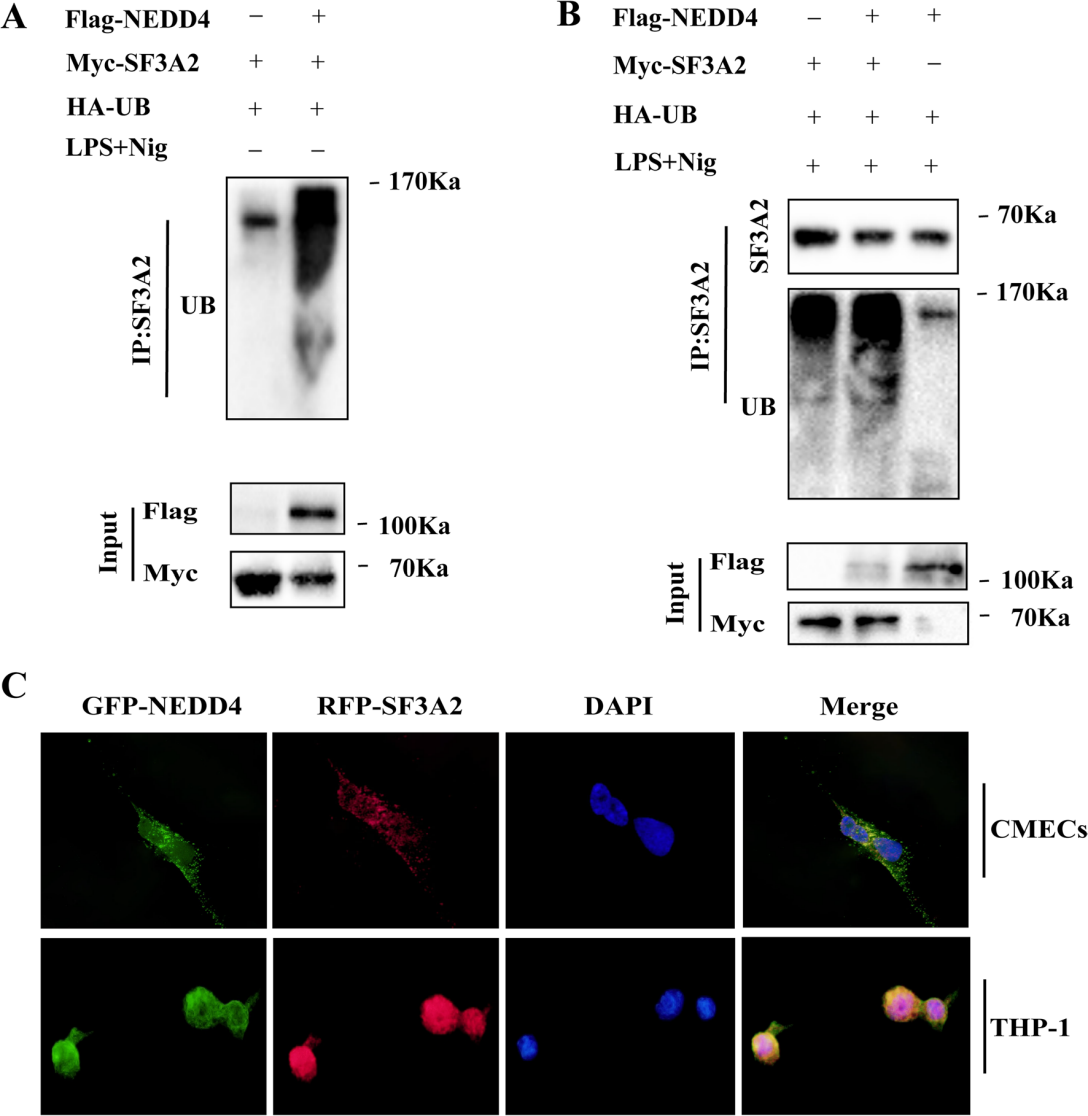
NEDD4 regulates SF3A2 ubiquitination indirectly inhibiting NLRP3 inflammasome activation. **A**, **B** HEK293T cells were transfected with Myc-SF3A2 and HA-UB, together with the empty control vector or Flag-NEDD4 expression vector. Cell lysates were immunoprecipitated with anti-RIP1 antibody after treatment with or without LPS and Nig stimulation. **C** CMECs transfected with GFP- and RFC-NLRP3 were fixed and incubated with a secondary antibody conjugated to Alexa Fluor 594 and Alexa Fluor 488. Colocalization between NEDD4 and NLRP3 was examined by fluorescence microscope

**Fig. 9:**
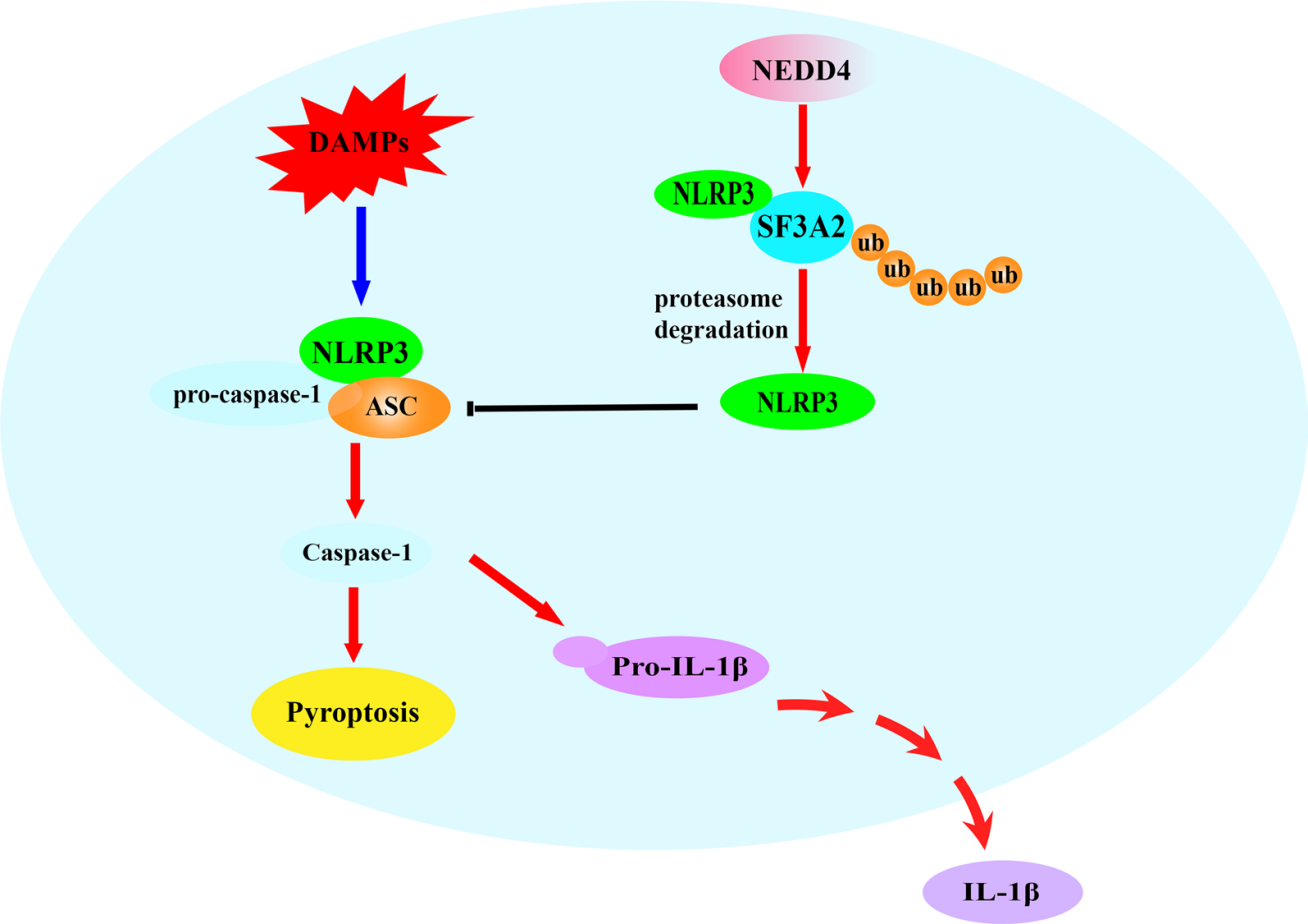
Schematic model for NEDD4 inhibiting NLRP3 inflammasome activation medicated pyroptosis. In macrophages, the constitutively expressed NEDD4 indirectly binds to NLRP3 through SF3A2, indirectly promotes proteasomal degradation of NLRP3 by regulating SF3A2 ubiquitination, and maintains NLRP3 low expression, which inhibits cell pyroptosis

We found no direct interaction between NEDD4 and NLRP3 (Fig. 5H); however, we did find that SF3A2 could directly interact with NLRP3 (Fig. 8A, B). To further investigate the underlying mechanism of NEDD4 regulating NLRP3 inflammasome medicated pyroptosis, we speculated that NEDD4 might exert its function by association with SF3A2 and might indirectly interact with NLRP3. To assess this hypothesis, we examined the colocalization between NEDD4 and SF3A2 in CMECs and THP-1 cells by immunofluorescence (Fig. 8C), finding ubiquitination of SF3A2 in 293 T cells overexpressing both NEDD4 and SF3A2, which was obviously lower than that in cells overexpressing only NEDD4 (Fig. 8A, B), thus indicating that NEDD4 promotes the ubiquitination of SF3A2 (Fig. 9).

## Discussion

In this study, we analyzed the effects of NEDD4 on the NLRP3 inflammasome activation mediated pyroptosis in vitro after an acute pro-inflammatory stimulus and in vivo in a MI/R mouse model. Previous studies suggested that E3 ubiquitin ligase NEDD4 is a key negative regulator for caspase-11 mediated pyroptosis in response to gram-negative bacterial infection and acute liver injury [22, 23]. But little is known about the function of NEDD4 on NLRP3 inflammasome activation induced pyroptosis during MI/R. In present study, we reveal that NEDD4 is a crucial negative regulatory component of the NLRP3 inflammasome pathway. Importantly, NEDD4 deficiency also promoted mouse death and inflammatory cell infiltration post-myocardial reperfusion or post-proinflammatory factor challenge. More interestingly, we also demonstrated that NLRP3 inflammasome activation induced inflammation and cell pyroptosis in macrophages are essential in regulation of the function on CMECs.

Accumulating evidence has been demonstrated that macrophage governs the fate of organs during inflammation and injury, including the heart [24, 25]. In the heart, deregulation of macrophages is a main cause of unrestrained inflammation to MI/R and is a critical factor in the pathogenesis of microvascular reperfusion injury. However, the role of macrophages signals in tailoring the function of CMECs remains unknown. To determine the effect of NLRP3 inflammasome activation in macrophages on CMECs, we observed that THP-1 cells-conditioned medium significantly induced the expression of C-GSDMD in CMECs and this trend was markedly augmented in THP-1 cells activated by the pro-inflammatory stimulus (LPS followed by Nig). To further assess the effect of NLRP3 inflammasome activation in THP-1 cells on CMECs, siRNA transfected THP-1 cells was used for further investigation, and found that THP-1 cells-conditioned medium treated with the LPS followed by Nig markedly suppressed the expression of C-GSDMD in CMECs. Based on these data, we draw a conclusion that NLRP3 inflammasome activation and pyroptosis in macrophages induced by pro-inflammatory stimulus acts as a key signal for promoting CMECs damage. Yet, the underlying mechanisms regulates NLRP3 activation medicated pyroptosis are not fully understood.

Ubiquitination is an important post-translational protein modification. Accumulated evidence suggested that ubiquitination of NLRP3 plays a key role in the regulation of inflammasome activation [26]. Our previous study has reported that NLRP3 inflammasome activation and pyroptosis which play a key role in the pathophysiology of microvascular injury following myocardial reperfusion [17, 27]. Therefore, we assume that the ubiquitination of inflammasome components precisely regulates NLRP3 inflammasome activity and pyroptosis in response to MI/R.

NEDD4 E3 ligase act as a critical role in a variety of cellular processes via the ubiquitination-mediated degradation of multiple substrates, and is well-known to play an important role in regulating the immune cell functions, including T cells, B cells and macrophage [22, 28]. Previous study has confirmed that the survival rate of NEDD4 deficiency mice after LPS stimulation is significantly decreased, and IL-1β in serum is markedly increased [29]. But whether and how NEDD4 plays a role in MI/R remains unknown. Here, by taking advantage of NEDD4 KO mice, the role of NEDD4 in MI/R was assessed. Surprisingly, post-myocardial ischemia reperfusion resulted in greater increased lethality, attenuated microvascular injury and myocardial infarct size in NEDD4 KO mice, indicating a novel role of NEDD4 in myocardial reperfusion injury. Previous reports have suggested that myocardial reperfusion injury is caused by myocardium or endothelium apoptosis and inflammation-induced cell necrosis with rapid recruitment of inflammatory cells in myocardial tissue [30, 31]. Here, we also detected increase inflammatory cells infiltration and inflammation in NEDD4 KO mice post-myocardial ischemia reperfusion or pro-inflammatory stimulus. In addition, we observed a higher level of IL-1β expression and GSDMD in NEDD4 KO mice.

As described previously, NEDD4 is a very important negative regulator of non-inflammasome activation and cell pyroptosis [22]. However, the impact of NEDD4 on NLRP3 inflammasome activation medicated-pyroptosis remains unclear. The in vitro data in our study also provided evidence that NEDD4 protects cells from pro-inflammatory stimulus induced NLRP3 inflammasome activation and pyroptosis in macrophages. To gain a deeper understanding of the underlying mechanism of NEDD4 in regulating NLRP3 inflammasome activation and pyroptosis in macrophages. We suppose that NEDD4 maintains the stability of NLRP3 by direct binding to NLRP3, and promotes the proteasomal degradation of NLRP3. Surprisingly, our data showed that NEDD4 promoted proteasomal degradation of NLRP3 but indirectly interacted with NLRP3. Thus, we speculated that NEDD4 might exert its function by association with such components. A recent study has shown that SF3A2 as new susceptibility gene for myocardial infarction [21].In our study, proteome and experimental data identified that SF3A2 could positively regulate NLRP3 inflammasome activation and cell pyroptosis. Meanwhile, we observed the colocalization of SF3A2 and NLRP3 in both the THP-1 cells and CMECs. Based on these data, we hypothesize that NEDD4 can bind to SF3A2 to indirectly mediate NLRP3 degradation, although this has not yet been reported. To support this hypothesis, the colocalization of NEDD4 and SF3A2 should further study. Interestingly, we found that SF3A2 interacted with E3 ligases of the NEDD4 and was ubiquitinated. Our data indicated that NEDD4 mediate NLRP3 degradation by ubiquitination SF3A2.

## Conclusion

In this study, we demonstrated that NEDD4 stabilized the NLRP3 to inhibit cell pyroptosis by promoting SF3A2 ubiquitination. Mechanistically, NEDD4 recruited the SF3A2 to remove the and prevented it from undergoing optineurin-mediated proteasome degradation. This study is valuable not only for increasing our understanding of the effect of THP-1 cells on CMECs, but also for demonstrating the potential of NEDD4 as a target for therapeutic interventions for inflammation-related cell pyroptosis.

## Clinical significance

Pyroptosis emerges as an important pathway for cell death induced by MI/R. In present study, it is of great clinical implication to elucidate the mechanism of E3 ubiquitin ligase NEDD4 through the degradation of NLRP3 preventing macrophage pyroptosis protects endothelial cell damage post-myocardial reperfusion, which thereby improve the treatment of patients with myocardial microvascular ischemia–reperfusion injury.

## Data Availability

The datasets supporting the conclusions of this article are included within the article files as well as materials prepared are available from the corresponding author on reasonable request.
